# Improving risk models for patients having emergency bowel cancer surgery using linked electronic health records: a national cohort study

**DOI:** 10.23889/ijpds.v9i5.2707

**Published:** 2024-09-18

**Authors:** Helen A Blake, Linda D Sharples, Jemma M Boyle, Angela Kuryba, Suneetha R Moonesinghe, Dave Murray, James Hill, Nicola S Fearnhead, Jan H van der Meulen, Kate Walker

**Affiliations:** 1 London School of Hygiene and Tropical Medicine; 2 Royal College of Surgeons of England; 3 University College London; 4 University College London Hospitals NHS Foundation Trust; 5 South Tees Hospitals NHS Foundation Trust; 6 Manchester Royal Infirmary; 7 Cambridge University Hospitals NHS Foundation Trust

## Objective

To investigate whether accuracy of a risk model for colorectal cancer (CRC) patients undergoing emergency surgery, including patient and tumour characteristics from disease-specific data, was improved by inclusion of physiological and surgical measures from linked treatment-specific data.

## Approach

Linked, routinely-collected data on patients undergoing emergency CRC surgery in England between December 2016 and November 2019 were used to develop a risk model for 90-day mortality. Backwards selection identified a 'selected model' of physiological and surgical measures in addition to patient and tumour characteristics. Model performance was assessed compared to a 'basic model' including only patient and tumour characteristics. Missing data was multiply imputed.

## Results

846 of 10,578 (8.0%) patients died within 90 days of surgery. The selected model included seven pre-operative physiological and surgical measures (pulse rate, systolic blood pressure, breathlessness, sodium, urea, albumin, and predicted peritoneal soiling), in addition to the ten patient and tumour characteristics in the basic model (year of surgery, age, sex, ASA grade, cancer site, number of comorbidities, emergency admission, TNM T stage, N stage and M stage). The selected model had considerably better discrimination than the basic model (C-statistic: 0.824 versus 0.783, respectively).

## Conclusion

Linkage of disease-specific and treatment-specific datasets allowed the inclusion of physiological and surgical measures in a risk model alongside patient and tumour characteristics, which improved the accuracy of predictions.

## Implications

Our new accurate and relatively simple risk prediction model for patients undergoing emergency CRC surgery will allow more accurate performance monitoring of healthcare providers and enhance clinical care planning.

